# Unexpected Evolution After Multivessel Coronary Artery Bypass Grafting in a Patient With Kawasaki Disease

**DOI:** 10.7759/cureus.15927

**Published:** 2021-06-25

**Authors:** Samjeris Victor, Kevin C McKillion, Jeffrey A Puette, Patrick McKillion, Matthew B Ellison

**Affiliations:** 1 Anesthesiology, West Virginia University, Morgantown, USA; 2 Pulmonary and Critical Care Medicine, Spectrum Health Medical Group, Michigan, USA

**Keywords:** kawasaki disease (kd), myocardial infarction, giant coronary artery aneursym, intracoronary thrombus, myocardial infarction, coronary artery angiography, coronary artery bypass grafting (cabg)

## Abstract

Kawasaki disease (KD), also known as mucocutaneous lymph node syndrome, is an acute vasculitis that frequently affects medium-sized blood vessels. The disease is usually self-limiting and most commonly affects children under five years of age. It often affects the coronary arteries and is the leading cause of acquired heart disease in developed countries. We report the case of a teenage boy who had a long-standing diagnosis of Kawasaki disease, underwent coronary artery bypass grafting surgery, and had a complicated medical course following the surgery.

## Introduction

Kawasaki disease (KD) is a vasculitis affecting small- to medium-sized vessels with a predisposition for affecting the coronary arteries [1]. It predominately occurs in children aged six months to five years and is more common among the Asian and Pacific Islander populations [1]. One of the most severe complications of KD is the development of coronary artery aneurysms (CAA), which can pose the risks of thrombosis and progressive stenosis. With thrombosis and stenosis of CAA, patients are at a higher risk for ischemic heart disease, myocardial infarction, and sudden cardiac death [2,3]. In the following case, a young male with a previous diagnosis of KD and giant CAA underwent multivessel coronary artery bypass grafting (CABG) surgery following myocardial infarction. Despite undergoing successful bypass surgery, the patient’s postoperative medical course was complicated by multiple additional episodes of chest pain and myocardial infarctions.

## Case presentation

**Figure 1:**
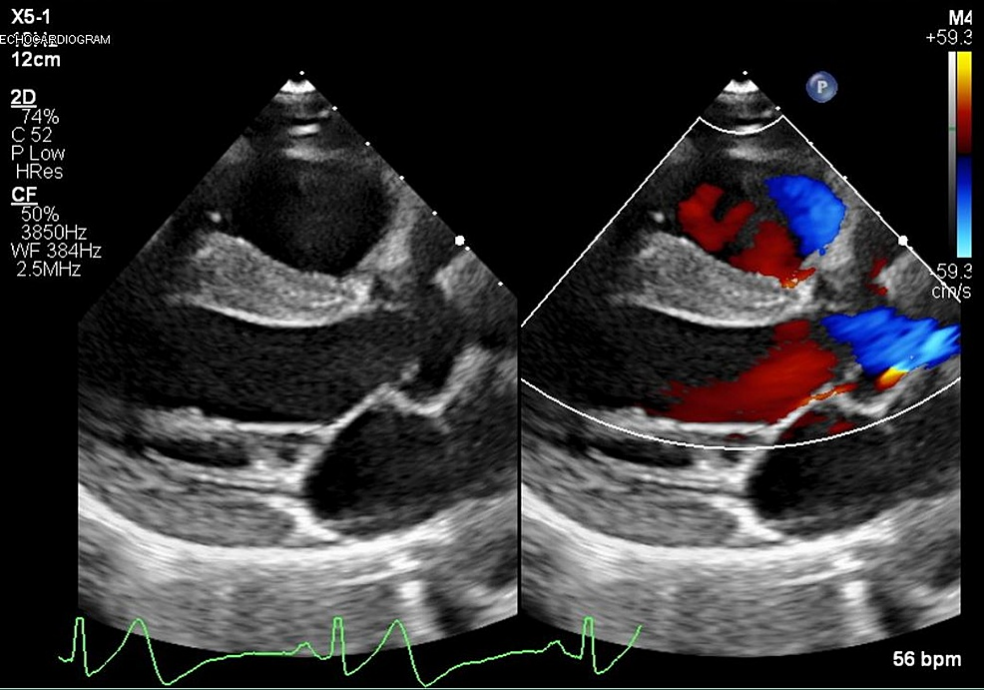
TTE-modified parasternal long-axis view demonstrating a dilated aortic root with giant right coronary artery aneurysm TTE, Transthoracic echocardiography.

**Figure 2:**
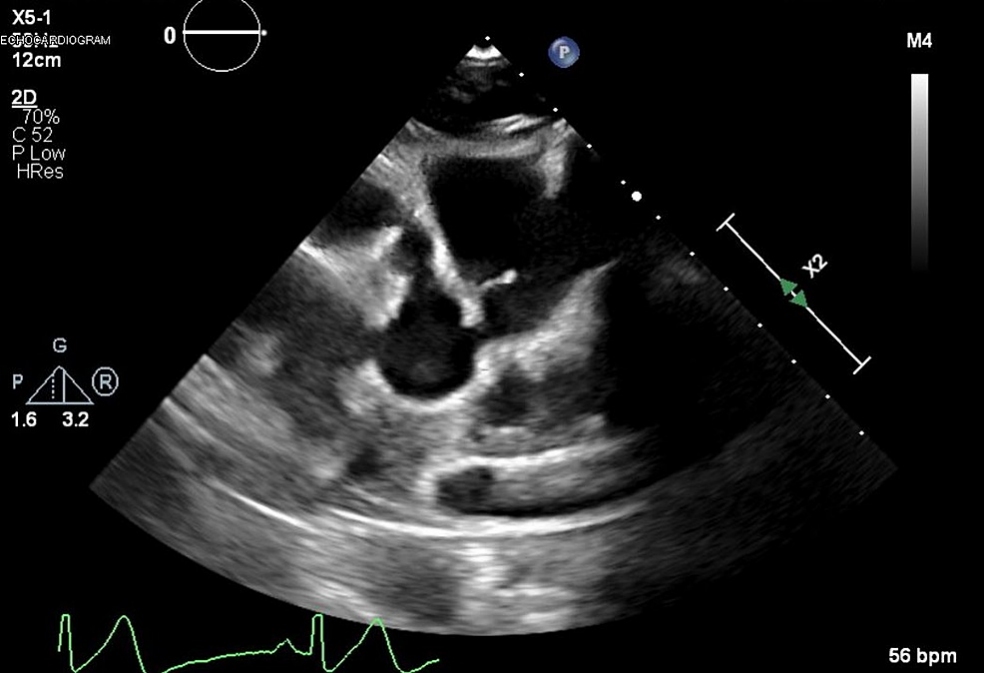
TTE-modified parasternal short-axis view demonstrating giant left and right coronary artery aneurysms TTE, Transthoracic echocardiography.

**Figure 3:**
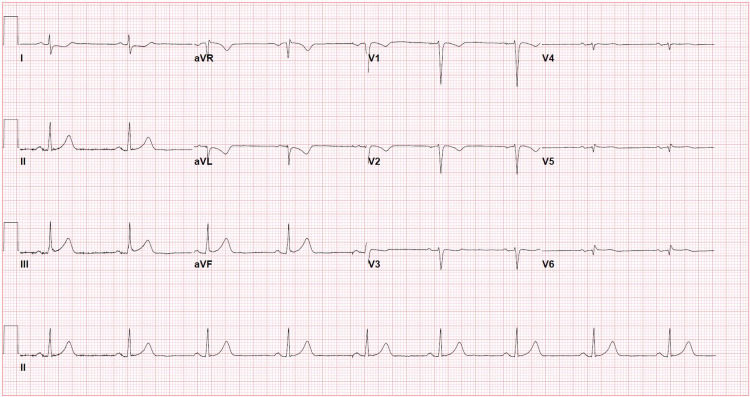
EKG demonstrating sinus bradycardia and ST elevation in the inferior leads EKG, Electrocardiogram.

A 17-year-old Caucasian male with known KD presented to the emergency department with new-onset chest pain. Upon arrival, his initial electrocardiogram (EKG) showed T-wave inversions with a serum troponin of 54 ng/L (normal range 0-30 ng/L) and a subtherapeutic international normalized ratio (INR) of 1.6. The patient's medications before arriving at the emergency department included metoprolol, aspirin 81 mg, clopidogrel (Plavix), and warfarin. He stated he was compliant with all his medications; however, he did not take any of them the morning of his current symptoms. He received a full-dose aspirin, and a pediatric cardiology service was consulted. Pediatric cardiology initiated a heparin infusion and performed a bedside echocardiogram, which demonstrated a dilated aortic root with giant right and left CAAs but normal left ventricular ejection fraction (LVEF) and contractility as shown in Figures 1, 2. A repeat EKG was performed confirming an ST-segment elevation myocardial infarction (STEMI) in the inferior leads as shown in Figure 3. After a team discussion that included family, both pediatric and adult cardiology, interventional cardiology, and the patient’s KD specialist at Boston Children's Hospital, Boston, USA, it was agreed that the patient should undergo emergent coronary angiography.

 

The patient was originally diagnosed with both KD and giant CAA in 2012. He receives follow-up and care from a KD specialist at Boston Children’s Hospital. Previously, in 2013, he underwent both cardiac magnetic resonance imaging (MRI) and coronary computed tomography angiography (CTA), which revealed thrombus throughout the right coronary artery (RCA) as well as a large left thrombus extending from proximal left anterior descending artery (LAD) to the descending portion. The coronary CTA showed multiple aneurysms including one in the left main coronary artery (measuring 22 mm x 21 mm x 30 mm), the proximal LAD (20 mm x 19 mm), and in the RCA (16 mm x 15 mm). Significant thrombus burden was noted in LAD and RCA without total occlusion of those vessels.

**Figure 4:**
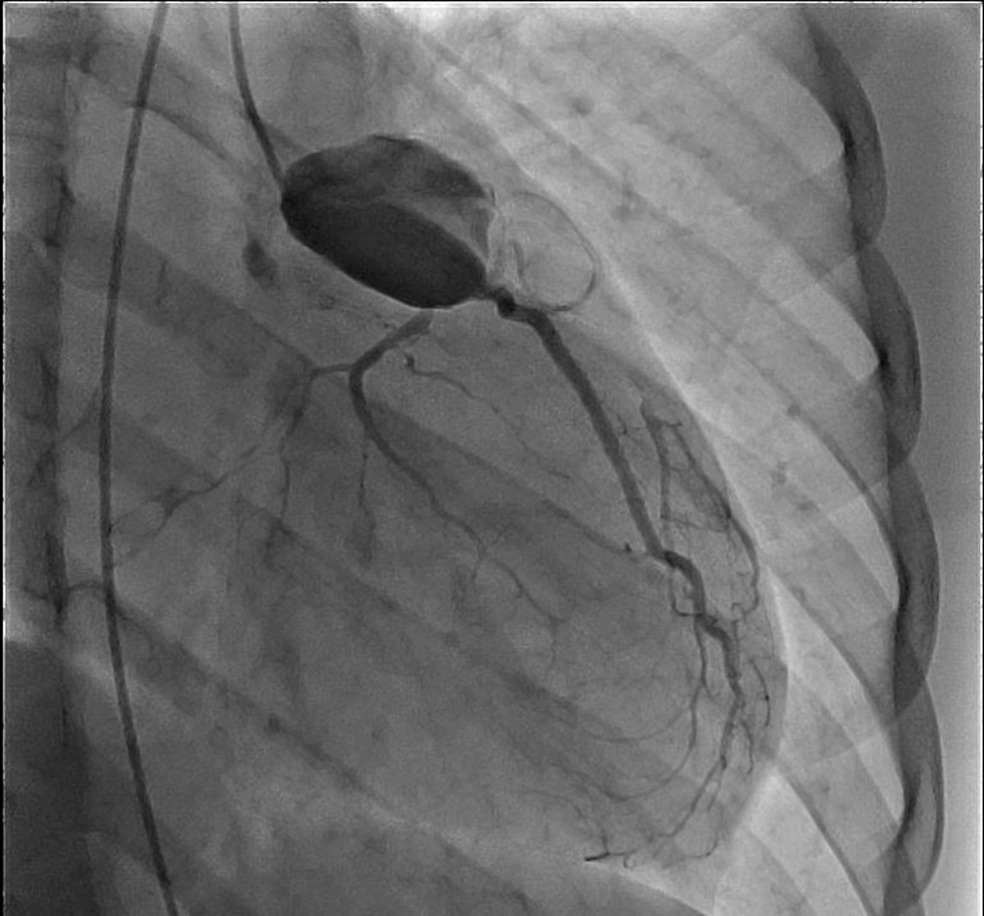
Coronary angiography demonstrating aneurysmal large left main with a concern for underlying dissection flap versus large layered thrombus. There is known occlusion of proximal left anterior descending artery.

**Figure 5:**
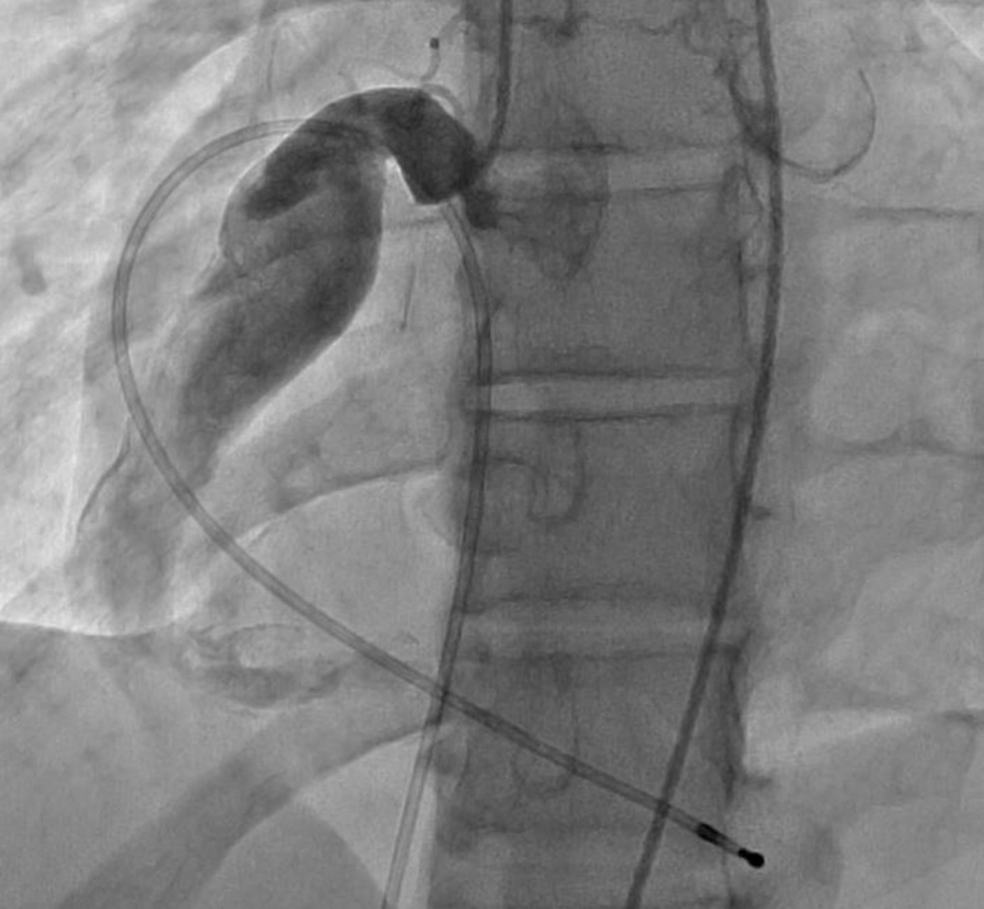
Coronary angiography demonstrating aneurysmal large right coronary with possible layered thrombus

**Figure 6:**
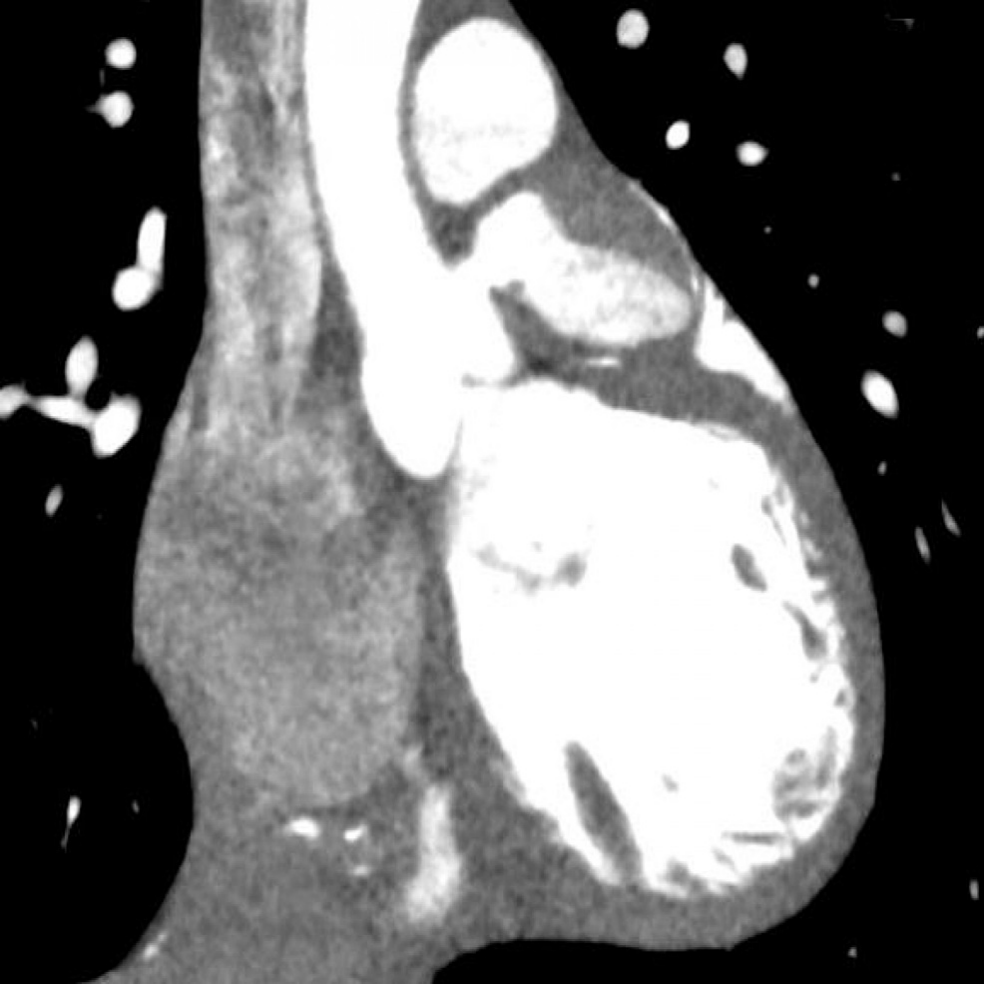
CTA demonstrating a giant saccular aneurysm of the left main coronary artery CTA, Computed tomography angiography.

During the current visit, emergent coronary angiography confirmed the previously aforementioned aneurysms and demonstrated a possible dissection of the left main coronary artery versus possible intracoronary thrombus, and no coronary intervention was performed. Images from the coronary angiogram are shown in Figures 4, 5. In order to better delineate the aneurysms and coronary arteries, a new CTA heart was obtained, which confirmed extensive thrombus burden in the left main (LM) coronary artery and RCA with total occlusion of LAD and the distal portion of RCA, thus confirming the diagnosis. An image from the CTA demonstrating a giant saccular LM CAA is shown in Figure 6. The patient was admitted to the cardiac intensive care unit and started on recommended medication regimen from the 2017 American Heart Association guidelines for KD revascularization, which included alteplase, aspirin, a heparin infusion, and an abciximab infusion [4].

 

**Figure 7:**
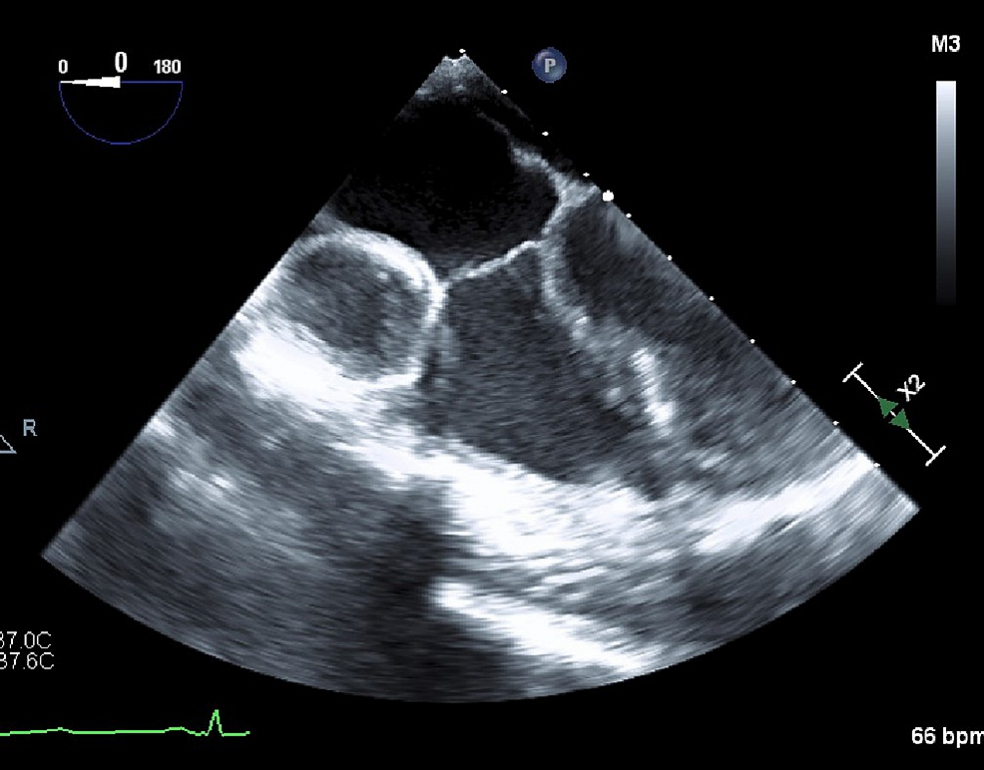
Mid-esophageal four-chamber view from intraoperative TTE demonstrating large aneurysm of right coronary artery with thrombus burden TTE, Transthoracic echocardiogram.

The cardiothoracic surgical service was consulted and evaluated in the patient for possible CABG surgery. After repeating the transthoracic echocardiogram (TTE) and trending the troponin level until it regressed (maximum was 39,000 ng/L), a multidisciplinary team determined that the patient should undergo bypass surgery. He underwent a successful two-vessel CABG on hospital day six. Because of his young age, both the right and left internal mammary arteries were utilized to bypass the RCA and LAD with no intraoperative complications. Figure 7 shows a giant right coronary aneurysm with a thrombus burden in the form of the intraoperative transesophageal echocardiogram. The patient’s postoperative course was unremarkable, and he was discharged home on the fourth day following his surgery. His medication regimen on discharge consisted of aspirin, statin, beta-blocker, and apixaban as per optimal medical therapy for secondary prevention of acute coronary syndrome (ACS) events with a scheduled follow-up three weeks later. Of note, the patient did not have a history of familial dysplasia nor had an abnormal lipid panel during this hospital course. The patient's C-reactive protein (CRP) and erythrocyte sedimentation rate (ESR) levels were also not checked at this visit nor did he receive any immunoglobulins or corticosteroids. 

Approximately four weeks after discharge, the patient presented to the emergency department (ED) with a new-onset chest pain radiating to the left jaw. Initial differential diagnosis included acute coronary syndrome, pulmonary embolism, and a possible postoperative complication. He admitted to missing multiple intermittent doses of apixaban, and thus a computed tomography scan with pulmonary embolism protocol was obtained but was negative for evidence of thromboembolic disease. A comprehensive workup was obtained including a repeat TTE, which was unremarkable, a troponin level (135 ng/L), an INR (subtherapeutic at 1.33), and serial EKGs, which showed persistent inverted T-waves in the inferior leads. Aspirin was administered, and both cardiology and cardiothoracic surgery were consulted. The patient was admitted and, along with aspirin, was given atorvastatin, heparin, and metoprolol. He underwent cardiac catheterization on day two, which showed that both bypass grafts were patent including the left internal mammary artery (LIMA) to LAD and the free right internal mammary artery (RIMA) coming off of LIMA graft. However, the free RIMA graft was 80% stenosed in the ostial to proximal segment due to a thrombus. With troponins trending down and pain improved, surgical intervention was avoided, and the patient was discharged on atorvastatin and metoprolol. He was also given a triple antithrombotic regimen that included aspirin, clopidogrel, and apixaban with a plan to discontinue the aspirin after one month and continue on the dual antithrombotic regimen indefinitely. Like his previous hospital visit, the patient's ESR and CRP levels were not checked nor did he receive any immunoglobulins or corticosteroids. He was asked to come for a follow-up four weeks after discharge.

Again, four weeks after his most recent visit, the patient presented back to the ED with a new-onset chest pain similar to that he experienced in his most recent admission. During this visit, his serum troponin was found to be significantly elevated at 4,939 ng/L; he was diagnosed with a non-ST elevation myocardial infarction (NSTEMI), and cardiology was consulted. He was quickly administered nitroglycerin, aspirin, clopidogrel, atorvastatin, and intravenous heparin and was admitted to cardiology in stable condition. A CTA was performed on hospital day two to evaluate RIMA graft patency based on the previously identified stenosis but identified no new abnormalities and patent bypass grafts. A TTE was also performed and demonstrated previously known basal inferior wall hypokinesis and previous inferior wall infarction with no new wall motion abnormalities. The patient was continued on all previously described medications and was admitted to the hospital overnight for continuous monitoring.

Overnight, he again experienced severe chest pain requiring morphine administration and a nitroglycerin infusion. A newly obtained EKG showed largely diffuse ST depressions, leading to cardiac catheterization, which was performed on the third hospital day. The catheterization showed aneurysmal dilation of all three major epicardial coronary arteries with a known significant thrombus burden following the CABG and total thrombotic occlusion of the mid-portion of the left circumflex (culprit vessel) at the distal edge of the aneurysmal segment. Troponin levels before catheterization were 1854 ng/L and levels post catheterization increased to 220,654 ng/L. The plan at the time was to continue with nitroglycerin, aspirin, and Plavix; increase the dose of atorvastatin; switch heparin to warfarin with an INR goal of 2.5-3.5, and monitor. On hospital day four, the patient exhibited new shoulder pain along with his chest pain. Limited TTE performed on hospital day four showed a newly decreased LVEF of 31% with known moderate mitral regurgitation. An EKG showed new ST depressions in leads V4-V6, and another urgent cardiac catheterization was performed. During catherization, angioplasty of the proximal portion of the first obtuse marginal branch was performed, and the patient received intracoronary tissue plasminogen activator (tPA) to the aneurysm of the left circumflex artery (LCX). He was started on an intensive anticoagulation/antiplatelet regimen that included heparin, tirofiban, and aspirin.

A cardiac MRI on hospital day five showed severely dilated left ventricle with an LVEF of 31%, multiple wall motion abnormalities, mitral regurgitation, and increased signal of the pericardium consistent with pericarditis. The patient was found to have elevated ESR (45 mm/hr) and CRP (169.7 mg/L), and due to the concern for hemorrhagic pericarditis, both the tirofiban and the heparin drip were discontinued. The patient was continued on high-dose aspirin (325 mg) and was started on colchicine for his newly diagnosed pericarditis. Like his two previous admissions, no immunoglobulins or corticosteroids were given during this hospital event.

The heart failure cardiology service was involved in this patient's care and recommended starting captopril with eventual transition to a longer acting renin-angiotensin-aldosterone system (RAAS) inhibitor (lisinopril) and metoprolol once the patient became more stable. They also noted if the patient were to become increasingly unstable, he would then be a candidate for temporal mechanical circulatory support as a bridge to definitive therapy. He was ultimately given a defibrillator vest secondary to his severely decreased left ventricular cardiac function and was discharged home on hospital day 11 and was asked to come for follow-up with cardiology within one week.

## Discussion

Giant CAAs in patients with KD are among the most serious complications associated with an acute myocardial infarction [4-6]. In a large patient series from Japan following KD patients, one of five patients subsequently died following a myocardial infarction (MI) [7]. In order to decrease the risk of CAA formation, the recommended treatment for a newly diagnosed KD patient is to administer high-dose intravenous immunoglobulin (IVIG) at 2 g/kg over 10-12 hours as well as adding high-dose aspirin (between 80 and 100 mg/kg/day) until the patient is afebrile for over 48 hours followed by six to eight weeks of low-dose aspirin (3-5 mg/kg) [8].

Giant coronary aneurysms (GCA) are defined as CAAs with an internal diameter greater than 8 mm. When GCA are larger than 8 mm, size regression is less likely and often progresses to myocardial ischemia and possibly death [2,8]. Percutaneous coronary intervention (PCI) and coronary artery bypass (CABG) are the two main procedures performed to establish coronary artery revascularization in patients with KD who illustrate signs of coronary ischemia [9]. The first known surgical treatment of Kawasaki coronary disease was performed by Kitamura et al. in 1974 where their patient underwent double-bypass surgery to the LAD and the RCA using autologous vein grafts [10]. However, these types of grafts were later shown to have poor long-term patency as well as limited growth potential as the patient aged [10]. Thus, the use of internal thoracic (mammary) artery grafts is now the conduit of choice in CABG operations for pediatric patients with Kawasaki coronary disease due to their excellent long-term patency and growth potential [10].

In a study of CABG versus PCI in KD patients, CABG patients were more likely to have complete revascularization and required less re-intervention than PCI patients [3]. In particular, the 2017 American Heart Association (AHA) guidelines recommend CABG over PCI in KD patients in older children and adults with KD and multivessel involvement [4]. The incidence of coronary events post-CABG was shown to be 13%, 19%, 30%, 38%, at 5, 10, 20, and 25 years, respectively [11]. One study that reviewed a series of patients who underwent single or multiple CABG for KD-specific coronary ischemia showed that cardiac events were observed 31 times in 25 patients who received a CABG [12]. Specifically, in that study, there was only one reported acute MI post-CABG. Based on the current literature, the most common reasons for cardiac events after CABG for KD include mammary graft stenosis at the anastomosis site, graft obstruction, fibroproliferative intimal thickening and atherosclerosis, late thromboses of other coronary arteries, and post-inflammatory progression of coronary artery disease [11].

The KD patient described in this case report underwent a CABG procedure following a STEMI and then proceeded to have two NSTEMIs post-CABG (all within the span of three months). Based on current literature, it is very uncommon for young patients with KD coronary disease to have serious cardiac events post-CABG procedure within such a short time frame. The CABG procedure that was performed for this patient, utilizing arterial grafts, has been documented as the safest and the most likely treatment to prevent post-surgical complications in KD patients. There were no reported complications intra-operatively for this patient's CABG surgery that would suggest a high risk of post-op complications like the NSTEMIs this patient experienced. Literature suggests that the approaches for secondary prevention after CABG procedures for atherosclerotic disease patients can be helpful for KD patients as well [13]. Following the patient's CABG surgery, he was prescribed aspirin for antiplatelet coverage, a statin for lipid management, and a beta-blocker for modulating excessive activation of the adrenergic nervous system that contributes to symptoms and pathophysiology of many cardiovascular diseases [4]. Even though the patient received and was reportedly compliant with these medications, he still developed multiple episodes of NSTEMIs. It is unclear whether this presentation was an active, uncontrolled status of KD; evidence to support this would be his RIMA graft stenosis and pericarditis. Even though they lack specificity for KD, ESR and CRP are common inflammatory markers used in assessing these patients. The patient presented here did not have his ESR and CRP levels assessed during his first two previously described hospital admissions. The patient also never received IVIG or corticosteroids during all three of his hospital admissions as there is not enough literature to support the use of these medications in KD patients presenting with symptoms associated with the acute coronary syndrome. Thus, this case is unique and demonstrates the need for additional research in order to understand, prevent, and treat serious complications in future KD patients.

## Conclusions

In this report, we present a 17-year-old male with KD and giant CAA with intracoronary thrombus burden who initially presented with a STEMI. After extensive testing and multidisciplinary planning, the patient successfully underwent a two-vessel CABG utilizing arterial conduits. However, even after successful revascularization, the patient continued to exhibit symptoms of angina and developed two additional NSTEMIs. In addition, further testing led to the diagnosis of systolic heart failure and pericarditis, leading to additional treatment and significant lifestyle modifications. This case highlights the importance of prompt treatment with IVIG and aspirin as soon as the diagnosis of KD is made in order to prevent the development of giant coronary aneurysm and associated sequela. Although KD is usually considered a self-limiting disease, this patient suffered debilitating long-term symptoms after successful coronary revascularization surgery. KD patients should be promptly treated by a specialist with experience in patients who have this condition and at a center that frequently cares for these patients.
